# Resistance to CymMV and ORSV in artificial microRNA transgenic *Nicotiana benthamiana* plants

**DOI:** 10.1038/s41598-018-28388-9

**Published:** 2018-07-02

**Authors:** Udomporn Petchthai, Celestine Shi Le Yee, Sek-Man Wong

**Affiliations:** 1 Department of Biological Sciences, National University of Singapore, Singapore, 119543 Singapore; 2 Temasek Life Sciences Laboratory, Singapore, 117604 Singapore; 3 National University of Singapore Research Institute in Suzhou, Jiangsu, 215123 P.R. China

## Abstract

Transgenic plants expressing artificial microRNAs (amiRNAs) have been shown to confer specific resistance to corresponding viruses. Here, we generated *Nicotiana benthamiana* transgenic lines containing *Oryza sativa* miR528 as backbone, expressing amiRNAs targeting RNA-dependent RNA polymerase (RdRp) gene of *Cymbidium mosaic virus* (CymMV) and *Odontoglossum ringspot virus* (ORSV). The amiRNA transgenic lines could express amiR-CymMV and confer high percentage resistance to CymMV, while lack of detectable level of amiR-ORSV expression in amiR-ORSV transgenic *N*. *benthamiana* plants led to weak resistance to ORSV infection. In this project, we provide the first report of CymMV-resistant transgenic *N*. *benthamiana* plants based on amiRNA strategy. We believe that this amiRNA approach can be extended to generate CymMV-resistant transgenic orchids.

## Introduction

Orchids are valued for their cut-flowers and potted plants in global floriculture markets due to their exquisite beauty and diverse floral patterns. Among the over 50 orchid-infecting viruses, there are two most prevalent and economically important viruses, namely *Cymbidium mosaic virus* (CymMV) and *Odontoglossum ringspot virus* (ORSV)^1^. CymMV and ORSV are single-stranded, positive-sense RNA viruses and are members of the Potexvirus and Tobamovirus groups, respectively. They can be transmitted mechanically via inoculation of infected plant sap, or through contaminated equipment such as cutting tools. In addition, these viruses are not transmitted by seeds or vectors^2,3^. CymMV induces chlorotic or necrotic patches on the leaves and the flowers of orchids. Furthermore, flowers infected with CymMV are deformed and displayed colour breaking. As for ORSV, it causes streaks or striped mosaic, diamond mottle or ringspots on orchid leaves and often results in ringspots or colour breaking in infected flowers^1,4^. Other than their natural hosts, CymMV and ORSV can infect *N*. *benthamiana* plant which is commonly used as a systemic host for plant virus research. CymMV causes short, white intermittent lines on leaves of CymMV-infected *N*. *benthamiana* plant. Mild mosaic and distorted emerging leaves are common symptoms induced by ORSV in *N*. *benthamiana* plant. Co-infection of these two viruses can be found in orchids cultivated in the field. Infection of both CymMV and ORSV caused *Cymbidium* orchid cultivars to display severe mosaic symptoms and necrosis^5^. Also, the viral RNA accumulation of both CymMV and ORSV increased when both viral RNAs were introduced into orchid protoplasts^6^.

RNA-mediated virus resistant transgenic plants have been successfully generated in several plant species with different RNA silencing strategies such as sense^7–9^, antisense^7,10,11^, double stranded^12,13^ and hairpin^14^ RNA. RNA silencing, as a result of the formation of double-stranded RNA (dsRNA), leads to sequence-specific gene silencing. These dsRNA of varying lengths, recognised by the enzyme Dicer which contains RNase III and dsRNA binding domains, are cleaved into short RNA duplexes, namely the short interfering RNAs and microRNAs^15,16^.

The emergence of artificial microRNAs (amiRNAs) provides a more specific targeting approach and has several advantages over other RNA silencing methods. The amiRNAs are designed based on the structure of an endogenous precursor miRNA and the endogenous miRNA region is replaced with desired miRNA sequence complementary to the target sequence. As the trans-acting plant miRNAs have high specificity, undesirable off-target effects can thus be avoided, allowing their silencing activity to be stably passed down to future generations^17–20^. In addition, the small size of amiRNA allows for the inclusion of multiple and unrelated amiRNAs within a single cassette which can then be used to create transgenic plant resistant to one or more viruses simultaneously^21–23^. It has been reported that the resistant effect of amiRNAs was more effective as compared to the ones obtained from short hairpin RNA^24^. Moreover, the environmental biosafety concerns of viral sequences that might complement or recombine with non-target viruses do not apply to amiRNAs^25^. The amiRNAs were used to effectively inhibit viral silencing suppressors such as helper-component proteinase (HC-Pro) of *Potato virus Y* and triple gene block 1 (TGBp1) of *Potato virus X*^26^.

Although there have been numerous achievements of virus resistant plants in previous studies^7–14,21–24,26^, the amiRNA approach against orchid viruses has not been reported. From the reported transgenic orchids, a few groups of researchers focused on the CymMV-resistant transgenic orchids by antisense approach to target the virus coat protein gene^27–29^. Here, the amiRNA approach was adopted to generate CymMV and ORSV resistant transgenic plants. We designed plant expression vectors pG0229, driven by the double *Cauliflower mosaic virus* (CaMV) 35S promoter, targeting the region of the gene coding for the RNA-dependent RNA polymerase (RdRp) of CymMV or ORSV. Due to the slow growth of orchids, *N*. *benthamiana* plant was selected to test the feasibility of the constructs. Here we showed that expression of CymMV-specific amiRNA hindered the infection of CymMV. However, ORSV-specific amiRNA transgenic *N*. *benthamiana* plants were weakly resistant to ORSV infection.

## Results

### Construction of dual viral resistance targeting RdRp of CymMV and ORSV

**Figure 1:**
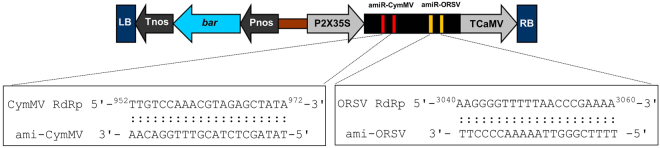
Design of amiRNA precursor pG0229-pre-amiRNA-CymMV-ORSV. LB, left border; RB, right border; Pnos, nopaline synthase promoter sequence; Tnos, nopaline synthase terminator sequence; *Bar*, bialaphos resistance gene; P2X35S, double Cauliflower mosaic virus 35S promoter sequence; TCaMV, Cauliflower mosaic virus terminator sequence; amiR-CymMV, amiRNA sequence of CymMV RdRp (in red) and it is followed by amiR-ORSV, amiRNA sequence of ORSV RdRp (in yellow).

The RdRp is crucial to viral replication. Thus, amiRNA construct targeting viral RdRp may confer stronger resistance of host plants against virus infection. The strategy was to identify suitable amiRNA targets by using Web MicroRNA Designer following the selection criteria^30^. By avoiding potential off-target sequences and low thermostability, the selected CymMV amiRNA sequence contained 21-nt complementary to nucleotides 952–972 of CymMV RdRp (5′-TATAGCTCTACGTTTGGACAA-3′). The ORSV amiRNA sequence selected was 5′-TTTTCGGGTTAAAAACCCCTT-3′, corresponding to nucleotides 3040–3060 of ORSV RdRp. After BLAST search, no *N*. *benthamiana* genes complementary to the 21-nt sequences of amiR-CymMV or amiR-ORSV was matched. These sequences replaced 21 nucleotides of mature miRNA in *Oryza sativa* miR528 (osa-miR528) precursor, inserted into a pG0229 vector to create a pG0229-preamiRNA-CymMV-ORSV construct (Fig. 1). Given our long-term goal to generate virus resistant transgenic orchids, we selected monocot conserved osa-miR528 precursor as a construct backbone. Based on a report on successful usage of amiRNA based on the osa-MIR528 precursor targeting *N*. *benthamiana UPF1* (Up-frameshift protein 1, accession number: EF187725.1) gene to silence *UPF1* transcripts^31^, we thus adopted the pre-miR528 backbone to produce amiRNA targeting RdRp RNAs of CymMV and ORSV.

### Verification of transgene integration using Southern blot analysis

**Figure 2:**
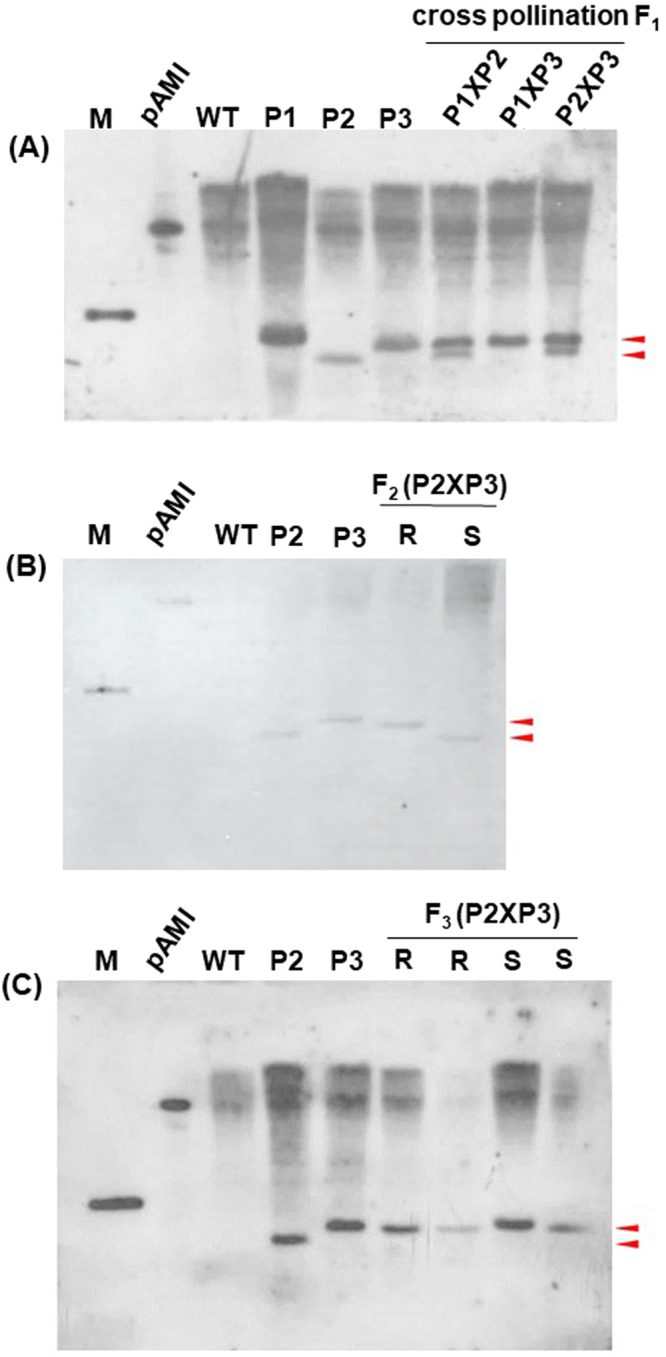
Confirmation of cross-pollination and stable insertion of the amiRNA transgene in transgenic *N*. *benthamiana* plants by Southern blot analysis. Aliquots (30 µg each) of *Eco*RI-digested plant total DNA were separated on a 0.8% agarose gel and transferred to a nylonmembrane which was hybridized with DIG-labeled amiRNA-CymMV-ORSV probe. M, GeneRuler^TM^ 1 kb Plus DNA Ladder; pAMI, pG0229-pre-amiRNA-CymMV-ORSV; WT, wild type; P, parental for cross pollination; R, resistant; S, susceptible. Red arrows depict the transgenes.

Attempts to combine transgenes into successive progenies by crossing difference transgenic lines were successful and verified by Southern blot. A total of eleven putative T_0_ transgenic lines were selected for testing of virus resistance. Positive T_0_ plants were self-crossed to produce T_1_ and then T_2_ transgenic plants. Three lines of T_2_ transgenic plants that showed symptomless to mixed infection of CymMV and ORSV were selected for crossing to produce F_1_ transgenic plants which contained the respective transgenes from both parents (P) (Fig. 2A). The transgene copy number of the F_2_ and F_3_ generation transgenic plants showed a single copy transgene (Fig. 2B and C).

### High percentage resistance to CymMV

**Figure 3:**
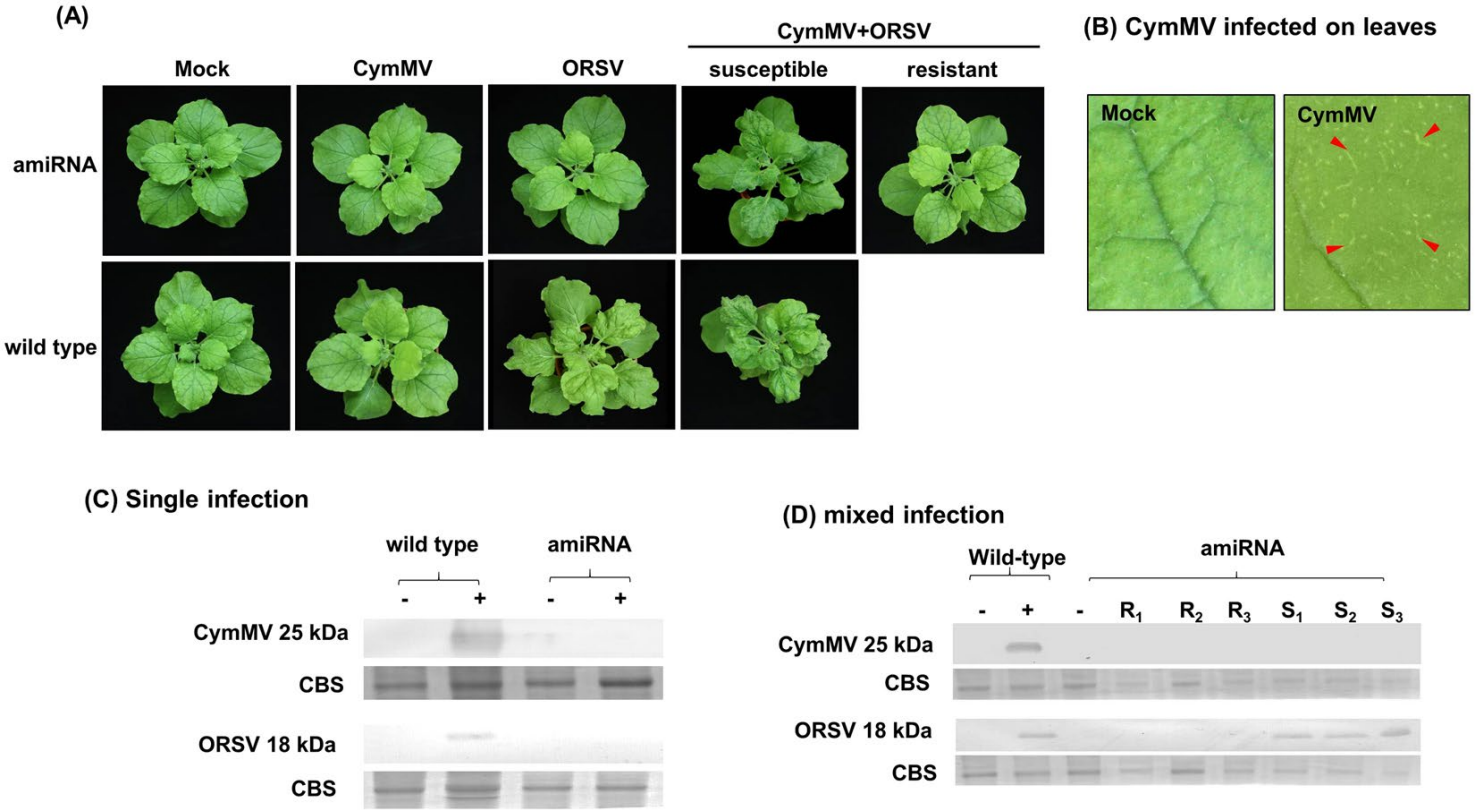
Analysis of resistance to CymMV or ORSV infection in transgenic *N*. *benthamiana* plants. (**A**) Symptoms of CymMV, ORSV and mixed infection in transgenic plants. Images were taken at 21 dpi. These transgenic plants showed no CymMV or ORSV symptoms. (**B**) CymMV infected wild-type *N*. *benthamiana* leaf showed short, white intermittent lines. (**C**) Transgenic amiRNA *N*. *benthamiana* plants inoculated singly with CymMV or ORSV showed no presence of CymMV or ORSV CP. Those plants inoculated with both viruses (**D**) showed absence of CymMV CP and ORSV CP in the resistant plants. Total plant proteins were extracted from upper uninoculated leaves of viral resistance amiRNA transgenic *N*. *benthamiana* plants. Wild-type plants were used as mock (−) and positive control (+), respectively. CBS is Coomassie Brilliant Blue staining of the large subunit of ribulose 1,5-bisphosphate carboxylase/oxygenase as a loading control. R_1–_-R_3_, resistant plants (symptomless) and S_1_–S_3_, susceptible plants (with viral symptoms).

To examine the CymMV resistance conferred by amiRNA-CymMV-ORSV in F_3_ and F_4_ generation transgenic *N*. *benthaniana* plants, wild-type and transgenic progenies harbouring amiRNA-CymMV-ORSV were challenged with CymMV at the six-leaf stage. At 21 days post inoculation (dpi), typical CymMV symptoms were observed in all wild-type plants (Fig. 3A and B). However, all amiRNA-CymMV-ORSV transgenic plants exhibited a high level of resistance, and no apparent disease symptoms was observed. The absence of virus in systemic leaves extracts of individually inoculated amiRNA-CymMV-ORSV transgenic plants at 21 dpi were confirmed by western blot (Fig. 3C).

### Weak resistance to ORSV

Although the same set of transgenic plants were inoculated with ORSV using wild-type *N*. *benthamiana* plants as controls at 8 dpi, most of the emerging leaves of amiRNA-CymMV-ORSV and all wild-type *N*. *benthamiana* plants showed distortion and mild mosaic symptoms. The infected plants showed more severe symptoms coupled with mosaic and curling of leaves at 21 dpi. F_3_ generation amiRNA-CymMV-ORSV transgenic plants showed 16% resistance to ORSV infection and majority of the plants displayed normal growth that was similar to mock-inoculated plants (Fig. 3A and Table 1). For F_4_ generation transgenic plants, all of them displayed symptoms of ORSV infection (Table 1). We postulate that failure to inhibit ORSV infection may be due to the lack of abundant small RNAs to silent the ORSV RdRp target or perhaps due to segregation of transgene.

**Table 1:** Percentage of resistant amiRNA-CymMV-ORSV transgenic *N*. *benthamiana* plants challenged with CymMV or ORSV inoculum.

Plants	Infectivity							
	CymMV				ORSV			
	F_3_		F_4_		F_3_		F_4_	
	I/T^a^	R (%)^b^	I/T	R (%)	I/T	R (%)	I/T	R (%)
amiRNA	0/12	100	0/19	100	10/12	16.6	18/18	0
WT	10/10	0	8/8	0	10/10	0	8/8	0

^a^I/T, Number of infected plants/number of total inoculated plants. ^b^R (%), percentage of resistant plants without symptoms after 21 dpi.

### Resistance of amiRNA transgenic plants to CymMV in CymMV + ORSV infection

To examine co-infection resistance conferred by amiRNAs in the transgenic *N*. *benthamiana* plants, the same batch of transgenic plants were inoculated with an equal amount of CymMV and ORSV at the six-leaf stage. At 8 dpi, distorted new leaves, as shown with ORSV infection, were observed in mixed infected plants. More severe leaf curling and stunted plant growth, as compared to mock-inoculated plants at 21 dpi, and typical CymMV and ORSV symptoms were observed in all wild-type *N*. *benthamiana* plants. For testing of virus resistance of amiRNA transgenic plants to mixed infection, some plants exhibited no symptom in co-infection, but most plants were susceptible and displayed ORSV symptoms. However, the symptoms of inoculated amiRNA transgenic plants were less severe than the wild-type *N*. *benthamiana* plants (Fig. 3A and Table 2). In the dot blot screening for the presence of CymMV and ORSV CP in tested F_4_ amiRNA transgenic plants, CymMV CP, but not ORSV CP, was not detected in most of the test samples (Supplementary Fig. S1). The resistant amiRNAs plants contained no CymMV and ORSV CP accumulation were verified by Western blot (Fig. 3D). CymMV CP was not detected in inoculated amiRNAs plants (R_1_–R_3_). ORSV CP was detected in inoculated amiRNA transgenic plants (S_1_–S_3_) (Fig. 3D).

**Table 2:** Percentage of resistant amiRNA-CymMV-ORSV transgenic *N*. *benthamiana* plants challenged with both CymMV and ORSV inocula.

Plants	Infectivity							
	F_3_				F_4_			
	CymMV		ORSV		CymMV		ORSV	
	I/T^a^	R (%)^b^	I/T	R (%)	I/T	R (%)	I/T	R (%)
amiRNA	1/42	97.62	40/42	4.76	5/19	73.68	16/19	15.78
WT	10/10	0	10/10	0	8/8	0	8/8	0

^a^I/T, Number of infected plants/number of total inoculated plants. ^b^R (%), percentage of resistant plants without symptoms after 21 dpi.

### Plants expressing amiR-CymMV effectively inhibited CymMV

**Figure 4:**
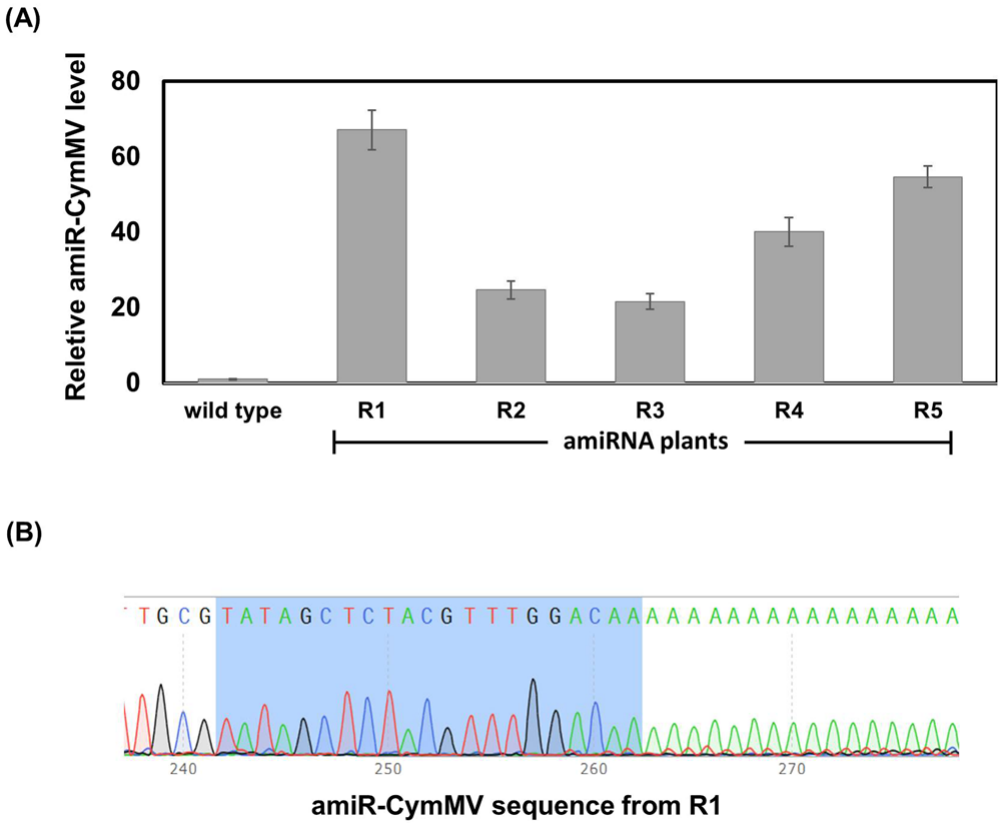
Validation of amiR-CymMV using the poly(A)-tailing based RT-PCR. (**A**) amiR-CymMV was detected in resistant transgenic plants inoculated with CymMV and ORSV. Error bars are standard deviations of three replicates. snRNA U6 was used as an internal control. (**B**) amiR-CymMV sequence (5′-TATAGCTCTACGTTTGGACAA-3′; highlighted in blue) was confirmed the correct amplicon by cloning and sequencing.

To further investigate the expression of amiR-CymMV or amiR-ORSV derived from pre-amiRNA-CymMV-ORSV, the poly(A)-tailing based real-time PCR was performed. The results showed that amiR-CymMV was detected in CymMV resistant F_4_ plants (R_1_–R_5_) with different expression levels of amiR-CymMV in individual transgenic plants and all expression levels of amiR-CymMV were sufficient to inhibit accumulation of CymMV RNAs (Fig. 4A). Cloning and sequencing results of real-time PCR products confirmed that they belonged to amiR-CymMV sequences (5′-TATAGCTCTACGTTTGGACAA-3′) containing a poly (A)-tail at the 3′ end of the sequence (Fig. 4B). On the other hand, expression level of amiR-ORSV was undetectable in transgenic plants, which was consistent with the appearance of non-resistant amiRNA plants showing obvious symptoms of ORSV infection.

## Discussion

Over the past two decades, advances in orchid transformation coupled with genetic engineering have gradually set the direction towards generating transgenic orchids^32^. From the reported transgenic orchids, most of them are research-based and gear towards optimisation of orchid transformation. There are reports of using sense or antisense RNA of CymMV coat protein to confer virus-resistance in orchids^27–29^ but those transgenic orchids have not been commercialized. As such, this project is an attempt to generate transgenic *N. be﻿nthamiana* plants as a proof of concept that can be extended to orchids. The amiRNA strategy was used to target two most prevalent orchid viruses CymMV and ORSV. Given the small size (21 nt) of amiRNA, the CymMV and ORSV amiRNAs were placed in a single cassette. This would confer resistance to CymMV and ORSV in the amiRNA transgenic plants generated.

We carried out screening for CymMV and ORSV resistance in T_0_ putative transgenic *N*. *benthamiana* plants (parental lines), based on symptoms observed and small RNA accumulation. However, no small RNA accumulation was detected in the plants screened. Based on the lack of symptoms, we know that some of the test plants are indeed virus resistant and we continued our crossing to produce more generations of plants for screening, despite we were unable to detect amiRNA from those plants. We detected accumulation of CymMV or ORSV coat protein (CP) by dot blot (Supplementary Fig. S1A).

We observed a lack of CymMV CP detected in the inoculated wild-type *N*. *benthamiana* plant sample (3F). However, the plant showed obvious symptoms of CymMV. We believed that the false negative result could be due to experimental errors in transferring the protein sample onto the membrane or during the protein extraction steps. We do not think it was due to failure of virus inoculation or virus detection sensitivity is poor. It does not reflect that the dot blot analysis used for virus resistance is not reliable.

Based on the results from the amiRNA transgenic *N*. *benthamiana* plants challenged with viruses, the tested transgenic plants indeed were resistant to CymMV, given the absence of symptoms and viral coat protein in the inoculated plants. Here, we showed that the amiRNA constructs targeting CymMV RdRp located at nucleotides 952–972 could inhibit CymMV accumulation effectively under single CymMV infection. The selected nucleotide sequences are located in the 5′-end of CymMV RdRp gene (~4 Kb), suggesting that initiation step of RdRp protein translation was blocked, thus preventing CymMV replication and gives rise to CymMV resistance.

Few ORSV transgenic plants tested were weakly resistant. Low percentage of F_3_ amiRNA transgenic plants confer resistance to ORSV inoculation, while no resistant was found in F_4_ plants. This might be due to segregation of the transgene in the F_4_ plants.

For the transgenic plants that were not resistant to ORSV could be explained with several possible reasons. The first reason is the designed sequence for suppression in amiRNA construction complementary to nucleotides 3040–3060 may not be the best choice. The sequences near the 3′-end of ORSV RdRp gene suggests that suppressing the gene of interest may not be as effective as sequences near the 5′-end. Target sequences nearer to the 5′ terminal may be more effective as the sequence inhibits translation of related protein at the beginning of translation.

ORSV RdRp genes encode two proteins (p126/p183 kDa) that are translated from the same open reading frame with a readthrough (nucleotides 63-3401/-3490). There are three functional domains identified in the RdRp. The first domain, from amino acids 72–287^33^, is a putative methyltransferase which is required for cap formation^34^. The second domain is a helicase from amino acids 820–1074^33^. The third domain is the polymerase domain defined by a GDD consensus sequence from amino acids 1372–1503^33^. ORSV is related to *Tobacco mosaic virus* (TMV), with genome similarity of 60%. The methyltransferase and helicase regions of TMV RdRp have been shown to be viral silencing suppressors^35^. Our design to target ORSV RdRp by amiRNA sequence did not include the methyltransferase region. Perhaps the region was translated and interfered with host RNAi pathway, resulting in replication of ORSV. From this result, it suggests that in order to suppress viral genes which encode multiple functional domains, in particular viral silencing suppressors, it is important to consider targeting multiple motifs in that particular viral gene^36^.

The second reason may be related to the free energy (ΔG) in structure folding of CymMV and ORSV amiRNA precursors, which are calculated as −38.50 kcal/mol and −34.90 kcal/mol, respectively (the original pre-miR528, ΔG is −46.20 kcal/mol). Since ORSV amiRNA precursor requires higher free energy to stabilise its stem-loop structure, less amiR-ORSV were generated. Moreover, it is possible that there is a problem in the junction usage of two amiRNA since the pre-amiRNA-ORSV backbone was placed after the pre-amiRNA-CymMV backbone. In this case, the pre-amiRNA-ORSV structure may not be folded properly and reduced the opportunity to generate mature sequence of amiR-ORSV, as shown in Supplementary Fig. S2, leading to less amiR-ORSV being produced and undetectable in amiRNA transgenic plants. This could be the reason why amiRNA transgenic plants confer weak resistance to ORSV infection. Recently *Oryza* pre-miRNA528 backbone-carried amiRNA has been shown to express less efficiently in *N*. *benthamina*^36^. On the other hand, our data demonstrate that *Oryza sativa* miR528 precursors harbouring amiRNA-CymMV-ORSV expressed in *N*. *benthamiana* plant can generate mature amiR-CymMV, allowing amiRNA transgenic plants to confer resistance against CymMV infection.

Northern blot analysis has been routinely used to validate the expression of small non-coding RNAs^37,38^. However, it requires a larger amount of RNA to be detected. Recently, a more direct and reliable PCR-based method for detection and quantification of all types of small non-coding RNAs has been developed^39,40^. This method provides many advantages such as only 200 ng total RNA is sufficient for cDNA synthesis and the accuracy of expressed amiRNA sequence has been proven^41^. Since small RNAs of amiR-CymMV and amiR-ORSV in F_1_-F_3_ plants were undetectable by Northern blot, we applied the poly(A) tailing based real-time PCR^41^ to detect small RNA in F_4_ plants, in which amiR-CymMV expression was detected and its sequence was verified (Fig. 4).

In conclusion, this is believed to be the first report of using amiRNA to generate CymMV-resistance transgenic *N*. *benthamiana* plants which were highly resistant to CymMV infection. We are optimistic towards the application of amiRNA as a promising approach to generate CymMV resistant orchids in future.

## Methods

### Construction of amiRNA-CymMV-ORSV

The sequence of CymMV and ORSV RdRp genes were analysed to predict target viral amiRNA in *N*. *benthamiana* genome [*Nicotiana benthamiana* EST NbGI-4.0 (GeneIndex)] by Web MicroRNA Designer (http://wmd3.weigelworld.org/cgi-bin/webapp.cgi). According to the criteria for selection of amiRNA^30^, the potential sequence without off-targets for CymMV amiRNA was 5′-TATAGCTCTACGTTTGGACAA-3′ and the ORSV amiRNA was 5′-TTTTCGGGTTAAAAACCCCTT-3′. The NW55 (miR528 *Oryza sativa*) was chosen as a backbone. The primers for construction of amiRNA-CymMV-ORSV were showed in Table S1. For obtaining the CymMV amiRNA fragments, three separate PCR steps were performed. First, using pNW55 as template with appropriate pairs of primers to generate the three DNA fragments. Primer C-5′ *Eco*RI was paired with primer C-II to obtain fragment C-A; primer C-I was used with C-IV to produce fragments C-B; primer C-III was used together with primer C-3′ to acquire fragment C-C. Another round of PCR was performed using the three fragments as template along with primers C-5′ *Eco*RI and C-3′ to generate a DNA fragment, C-amiRNA. To obtain the ORSV amiRNA fragment, similar PCR steps were performed with appropriate primers as follows: O-5′/O-II; O-I/O-IV; O-III/O-3 *Bam*HI′; O-5′/O-3 *Bam*HI′. Using both CymMV and ORSV amiRNA fragments as template with primer CO/C-5′ *Eco*RI/O-3 *Bam*HI′, a fused DNA fragment of CymMV-ORSV amiRNA was obtained for subsequent cloning. The 522 bp fragment was inserted into a binary vector pG0229 to become pG0229-preamiRNA-CymMV-ORSV construct (Supplementary Fig. S3).

### Transformation of pG0229-pre-amiRNA-CymMV-ORSV to *N*. *benthamiana* plant

*N*. *benthamiana* plants were transformed with pG0229-pre-amiRNA-CymMV-ORSV construct by *Agrobacterium*-mediated leaf disc method^42^. The primary transformants (T_0_) were first screened by PCR for the presence of the respective transgenes. PCR-positive plants were allowed to self-pollinate, the seeds were collected as T_1_. Basta® resistance T_1_ lines expressing plants exhibiting virus resistance were harvested for seeds. T_2_ progenies of CymMV and ORSV resistant lines were subsequently crossed to gain more transgene copy number.

### Plant materials and virus inoculation

Wild-type and transgenic *N*. *benthamiana* plants were grown at 24 °C under a 16 h/8 h photoperiod. Plants were mechanically inoculated with 100 ng of purified CymMV, ORSV, and mixed infection of CymMV + ORSV. For negative control, 0.01 M phosphate buffer (pH 7.0) was used and wild-type *N*. *benthamiana* plants were inoculated with CymMV, ORSV, and mixed infection of CymMV + ORSV functioned as positive control. Symptoms were observed 21 days after virus inoculation to select for viral resistant and susceptible plants.

### Cross pollination

Cross pollination (Supplementary Fig. S4) was performed on T_2_ transgenic *N*. *benthamiana* plants containing amiRNA-CymMV-ORSV transgene by using young flowers from two amiRNA-based lines (A and B). Stamens were removed from one flower (C) and pollen was obtained from the other flower (D) and transferred to the stigma of the flower without stamens (E and F). A developing seed pod was observed a week after successful cross pollination (G) and the seeds can be sown one month post cross pollination, giving rise to crossed F_1_ transgenic plants.

### Determination of transgene copy number by Southern blot

Genomic DNA of *N*. *benthamiana* leaves (30 µg) were extracted according to the cetyltrimethyl ammonium bromide (CTAB) extraction protocol^43^ and subsequence to digest with *Eco*R1 (Thermo Scientific). *Eco*RI-digested DNA was separated on a 0.8% agarose gel and the blotted membrane was hybridized with DIG-labeled specific probe, complementary to the sequence of the transgene pre-amiRNA-CymMV-ORSV prepared by PCR DIG Probe Synthesis Kit (Roche). Southern blot was performed according to DIG Application Manual for Filter Hybridisation (Roche). All blots were scanned by Canon scanner LiDe210.

### Determination of viral coat protein by western blot and dot blot

Total proteins of viral resistant and susceptible plants were extracted after 21 days post inoculation and used for western blot and dot blot. Leaf samples were homogenised at a ratio of 1:10 of plant material to protein extraction buffer (0.22 M Tris-Cl (pH 7.4), 250 mM sucrose, 50 mM KCl, 1 mM MgCl_2_). To detect CP of CymMV or ORSV by western blot, total proteins separated by 12% SDS-PAGE were electroblotted onto a nitrocellulose membrane (Bio-Rad Laboratories). The membrane was incubated in primary rabbit antibody containing anti-CymMV CP or anti-ORSV CP which produced following protocol by to Eun & Wong^44^ and followed with secondary antibody, anti-rabbit immunoglobulin G conjugated with alkaline phosphatase (Sigma, St. Louis). Then, colourimetric detection was performed with nitro blue tetrazolium and 5-bromo 4-chloro 3-indolil phosphate. All blots and gels were scanned by Canon scanner LiDe210. For screening of the presence of CymMV or ORSV CP by dot blot, 3.5 µl of sample was carefully deposited onto nitrocellulose membrane (Bio-Rad Laboratories), air-dried for 5 min, then followed by Western blot.

### Small RNA detection by poly(A)-tailing based real-time PCR

One µg of low molecular weight RNA was used to validate small RNA products of amiRNA-CymMV-ORSV following the instruction of All-in-One™ miRNA qRT-PCR Detection (GeneCopoeia™). The specific real-time PCR primers for detecting amiR-CymMV and amiR-ORSV were 5′-GCGTATAGCTCTACGTTTGGAC-3′ and 5′-GCGTTTTCGGGTTAAAAACCCC-3′, respectively. snRNA U6 (5′-GCGGGAACGATACAGAGAAGATTAGC-3′) was used as an internal control.

All data are available for checking and verification.

## Electronic supplementary material

Supplementary Information
