# Supplementary material for: Resistance to CymMV and ORSV in artificial microRNA transgenic Nicotiana benthamiana plants
Source: Sci Rep. 2018 Jul 2;8:9958. doi: 10.1038/s41598-018-28388-9 (PMC6028384; doi:10.1038/s41598-018-28388-9)
Supplement: Supplementary file 1 — Supplementary Information [file 41598_2018_28388_MOESM1_ESM.pdf]

Resistance to CymMV and ORSV in artificial microRNA transgenic *Nicotiana benthamiana* plants

Udomporn Petchthai[1], Celestine Yee Shi Le[1], and Sek-Man Wong[1, 2, 3*]

[1]Department of Biological Sciences, National University of Singapore, Singapore

[2]Temasek Life Sciences Laboratory, Singapore

[3]National University of Singapore Research Institute in Suzhou, Jiangsu, PRC

[*]Corresponding author: Sek-Man Wong; Email: dbswsm@nus.edu.sg

Phone: +659856643; Fax: +6567792486

Udomporn Petchthai E-mail: dbsup@nus.edu.sg

Celestine Shi Le Yee E-mail: celestineysl@gmail.com

Supplementary information

Supplementary Table S1. Primers for construction of amiRNA-CymMV-ORSV

Name of primers	Sequence of primers (5' to 3')
C-I	agTATAGCTCTACGTTTGGACAAcaggagattcagtttga
C-II	tgTTGTCCAAACGTAGAGCTATActgctgctgctacagcc
C-III	ctTTGTCGAAAGGTAGAGCTATAttcctgctgctaggctg
C-IV	aaTATAGCTCTACCTTTCGACAAagagaggcaaaagtgaa
O-I	agTTTTCGGGTTAAAAACCCCTTcaggagattcagtttga
O-II	tgAAGGGGTTTTTAACCCGAAAActgctgctgctacagcc
O-III	ctAAGGGCTTTATAACCCGAAAAttcctgctgctaggctg
O-IV	aaTTTTCGGGTTATAAAGCCCTTagagaggcaaaagtgaa
C5’ *Eco*RI	tcgaattccagcagcagccacagcaaa
C3'	ttctacttatcacccgaggggatc
O5'	gatttcgtgtacggtgttaatttcacc
O3’ *Bam*HI	cgcggatccgctgctgatgctgatgccat
CO	ggctgctgctgcccgggggattccccggggctgctgatgc

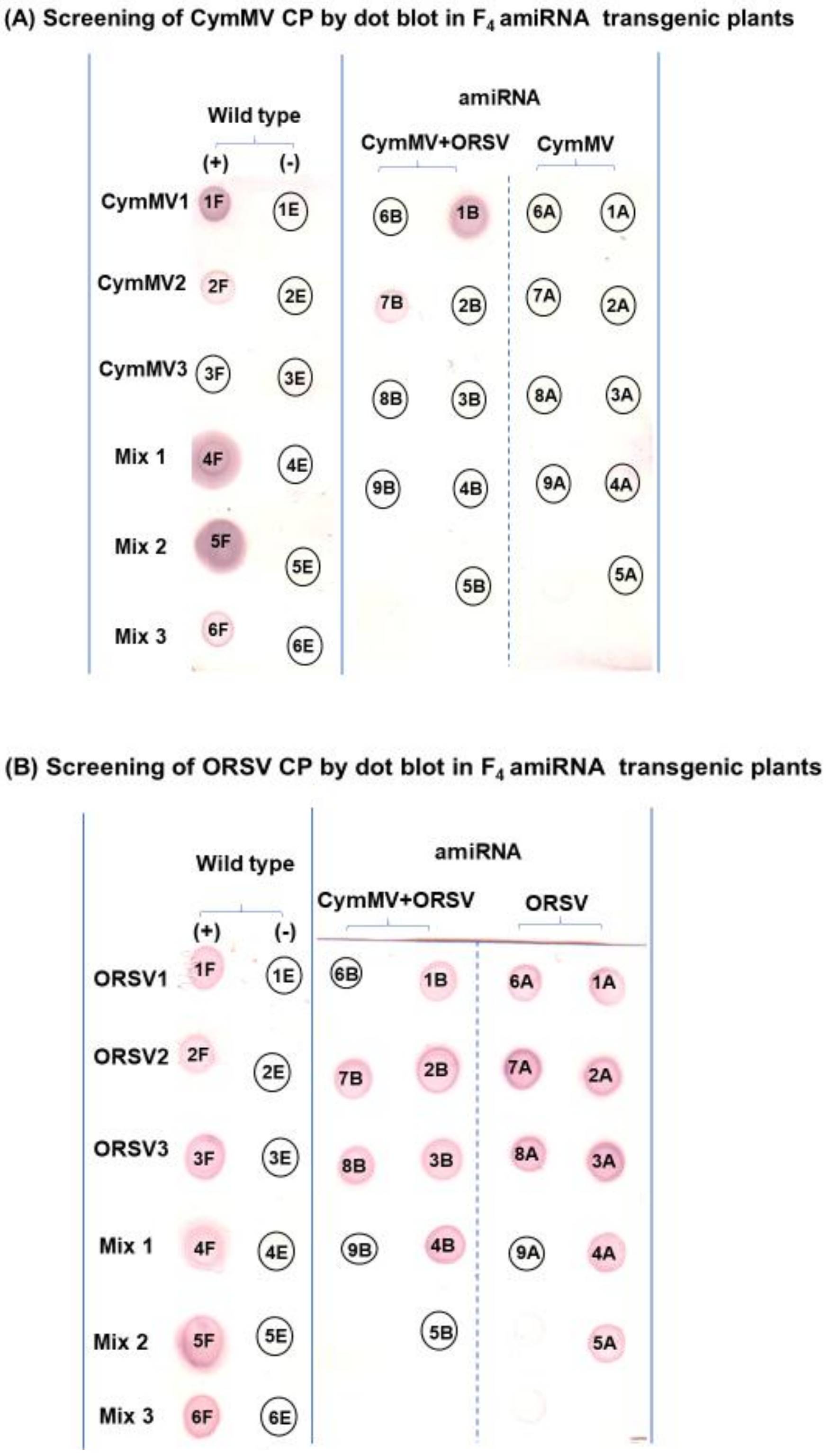

Supplementary Figure S1. Screening of CymMV coat protein (CP) (A) and ORSV CP (B) accumulation in single (CymMV or ORSV) and mixed (CymMV+ORSV) infected *N.*

benthamiana plants by dot blot. Circles represent samples with no viral CP detected. Sample numbers indicate different plants screened. CymMV CP was not detected in the inoculated wild-type *N. benthamiana* plant sample (Panel A CymMV3 3F). This is believed to be an artefact because the test plant showed typical symptoms induced by CymMV (see explanation in Discussion).

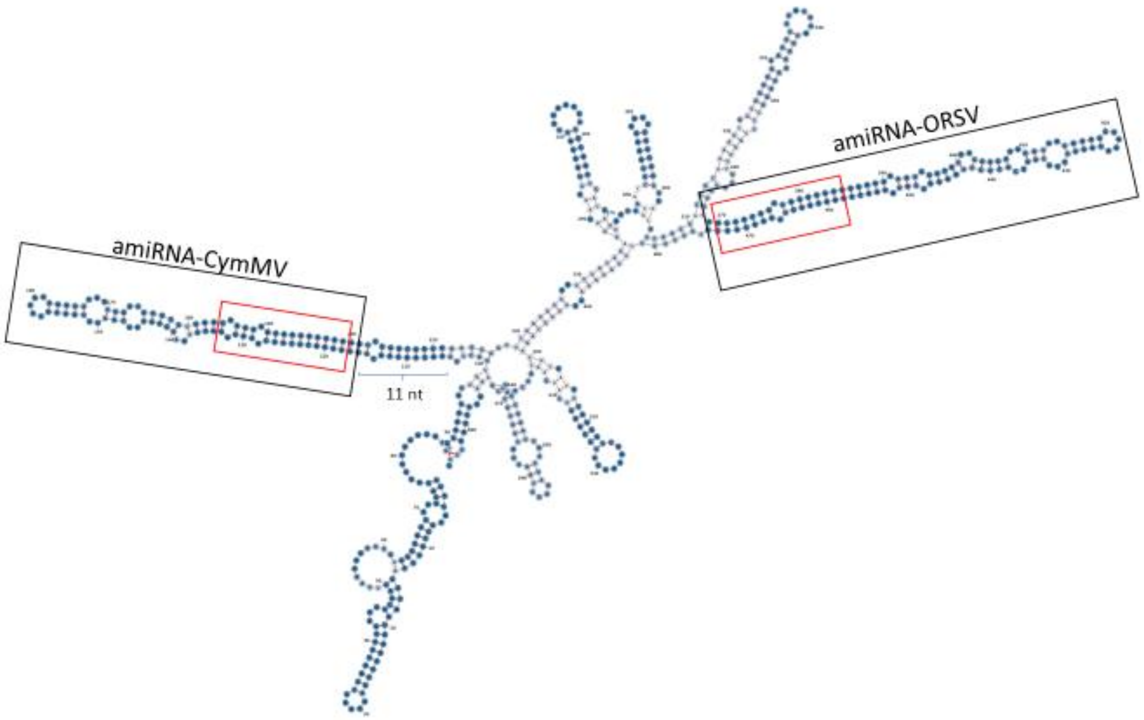

Supplementary Figure S2. Predicted folding structure of pre-amiRNA-CymMV-ORSV analyzed by RNAfold WebServer (http://rna.tbi.univie.ac.at/cgi-bin/RNAWebSuite/RNAfold.cgi). The stem-loop structures of amiR-CymMV and amiR-ORSV are indicated in the black rectangles. The mature sequence of amiR-CymMV and amiR-ORSV are in the red rectangles.

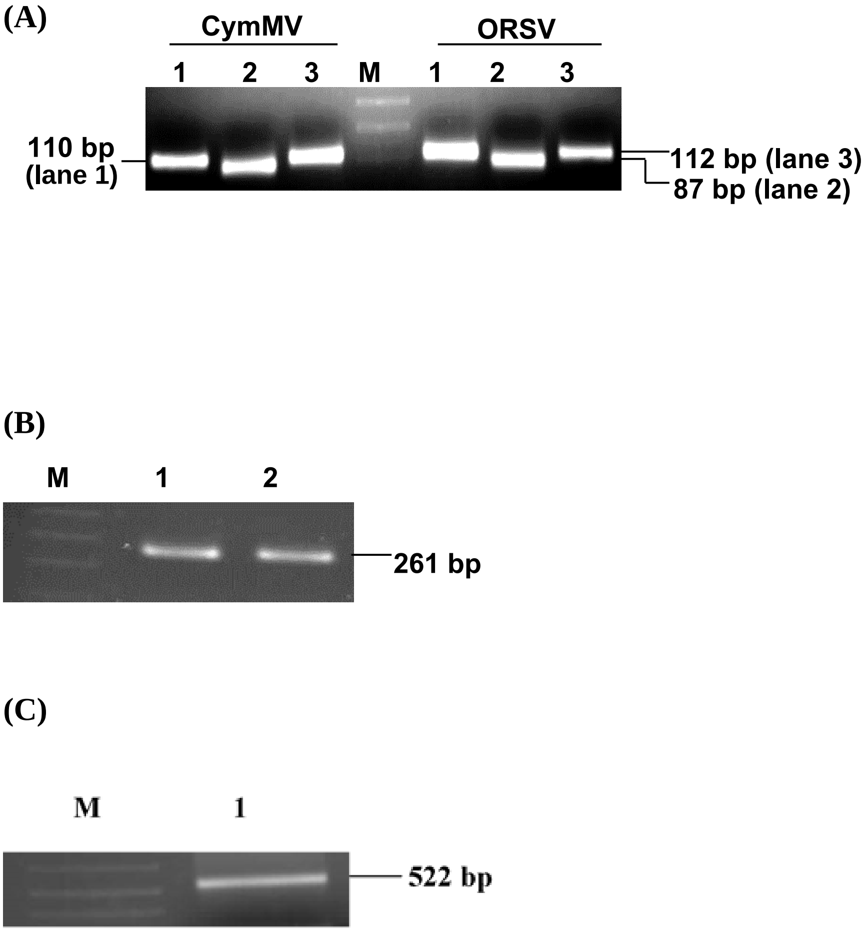

Supplementary Figure S3. Construction of pG0229-preamiRNA-CymMV and ORSV
(A) Using pNW55 as plasmid template, site directed mutagenesis was performed to include amiRNA and amiRNA* sequences of CymMV and ORSV to obtain products A (lane 1), B (lane 2), and C (lane 3), respectively. Lane M represents 1 kb DNA ladder.
(B) After obtained products A, B, C for CymMV and ORSV, respectively, the products were pooled individually to obtain amiRNA precursors for CymMV (lane 1) and ORSV (lane 2), respectively. Lane M represents 1 kb DNA ladder.
(C) amiRNA precursors of CymMV and ORSV were used as template to generate amiRNA precursor of CymMV-ORSV (lane 1) by PCR. Lane M represents 1 kb DNA ladder.

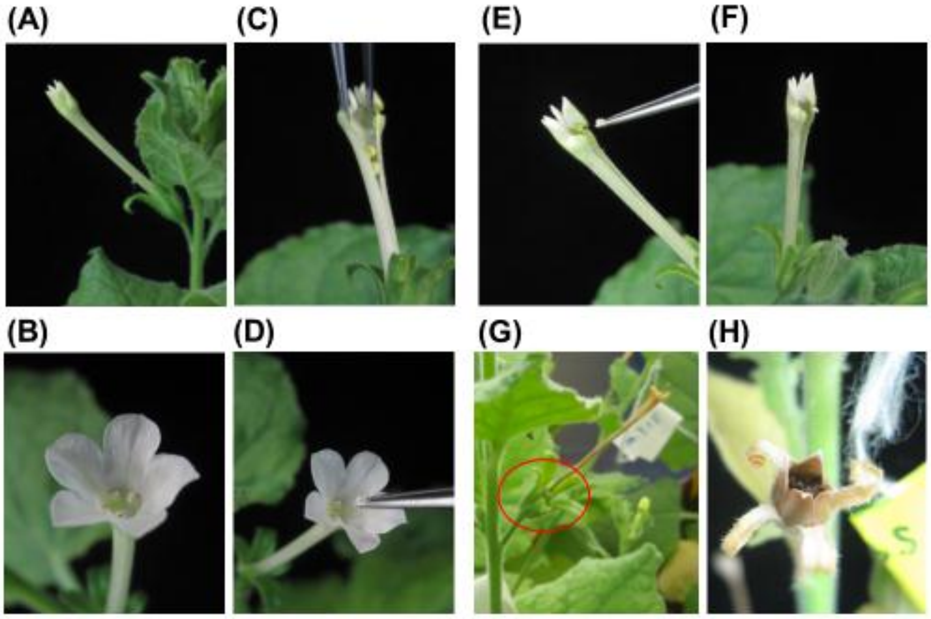

Supplementary Figure S4. Cross pollination process to generate F_1 transgenic plants.

Cross pollination was performed on T_2 transgenic *N. benthamiana* plants containing amiRNA-CymMV-ORSV transgene by using young flowers from two amiRNA-based lines (A and B). Stamens were removed from one flower (C). Pollen was obtained from the other flower (D) and transferred to the stigma of the flower without stamens (E and F). A developing seed pod was observed a week after successful cross pollination (in red circle). (G) Seeds were sown one month post cross pollination, giving rise to crossed F_1 transgenic plants.
